# Impact of childhood burn injuries on breastfeeding: a case report

**DOI:** 10.1186/s13006-019-0210-4

**Published:** 2019-04-18

**Authors:** Zurraini Arabi, Ezura Madiana Md Monoto, Agusmanan Bojeng

**Affiliations:** 1 Department of Family Medicine, Faculty of Medicine and Health Sciences, Universiti Malaysia Sarawak, Kota Samarahan, Sarawak Malaysia; 2 Department of Family Medicine, Faculty of Medicine, Universiti Kebangsaan Malaysia, Kuala Lumpur, Selangor Malaysia; 3 Department of Radiology, Faculty of Medicine and Health Sciences, Universiti Malaysia Sarawak, Kota Samarahan, Sarawak Malaysia

**Keywords:** Childhood burns, Breastfeeding

## Abstract

**Background:**

Pre-pubescent girls with chest burns are at risk of complication associated with scarring and impairment in breast development. This case illustrates how burn injuries in childhood in a first-time mother have affected her in terms of breastfeeding.

**Case presentation:**

In May 2015, a 20 year old first-time mother at 36 weeks gestation was seen in a district health clinic in Kuching, Sarawak in regards to her ability to breastfeed. She had a history of a flame burn at the age of 5 years old to her chest, abdomen, upper limb and part of her trunk. A skin graft was done on her whole chest and abdomen. Despite the injuries, she had pubertal and antenatal breast development. Her abdomen was able to stretch to accommodate her pregnancy. Physical examination showed a pregnancy which corresponded to date. The skin over her chest and abdomen appeared tight with areas of hyperpigmentation and hypopigmentation due to scarring from the skin graft. Breast tissues were palpable over her chest. The areola and nipple tissue were completely absent with complete scarring of the nipple-areolar complex. There was no duct opening to the areola for milk expression or leakage. Counselling regarding her breastfeeding issues was done. She delivered her baby at full term via spontaneous vaginal delivery with no complication. Oral cabergoline 1 mg was given on the first day postpartum. The baby was given infant formula via bottle feeding as the feeding method of choice.

**Conclusion:**

Counselling plays an important part in the management of a mother with breastfeeding difficulty. Allaying the possible guilty feelings of not being able to breastfeed will fulfil the emotional gap which may arise in a mother with these challenges.

## Background

Burns are an important cause of morbidity and mortality in children. Burns are the only type of unintentional injury where females have a higher rate of injury than males. Worldwide, the fire related death rate for girls is 4.9/100000 population and 3.0/100000 for boys [1]. In all burn injuries, nearly a quarter occur in children under the age of 16, of who the majority are under the age of five [2].

Pre-pubescent girls with chest burns have a significant risk of long-term problems associated with scarring and breast development [3]. Burns involving the anterior chest wall may cause injuries to the breast and the nipple-areolar complex which can potentially impair and/or destroy both function and aesthetics [4]. Lactation may still occur after pregnancy as loss or distortion of the nipple-areolar complex due to burn does not necessarily indicate that the underlying gland will not develop completely [4]. Damage to the breast is particularly important to the prepubescent young girl as it could lead to psychological issues due to potential loss of femininity [5]. Issues regarding breastfeeding may have never been considered until they reach the antenatal stage as a preparation of their parenting choice.

Breastfeeding is promoted worldwide because of the importance for infant and maternal health [6]. Women who are unable to breastfeed can experience a sense of guilt due to their inability of giving what is known to be the normative standard of infant feeding and nutrition for their child. These negative feelings can impact on how they adapt and their enjoyment towards motherhood [7].

This case illustrates a young first-time mother who had a skin graft done over her chest and abdomen due to burn injuries in childhood and how it has affected her in terms of breastfeeding. The clinical presentation and management of this patient are described as follows.

## Case presentation

**Fig. 1:**
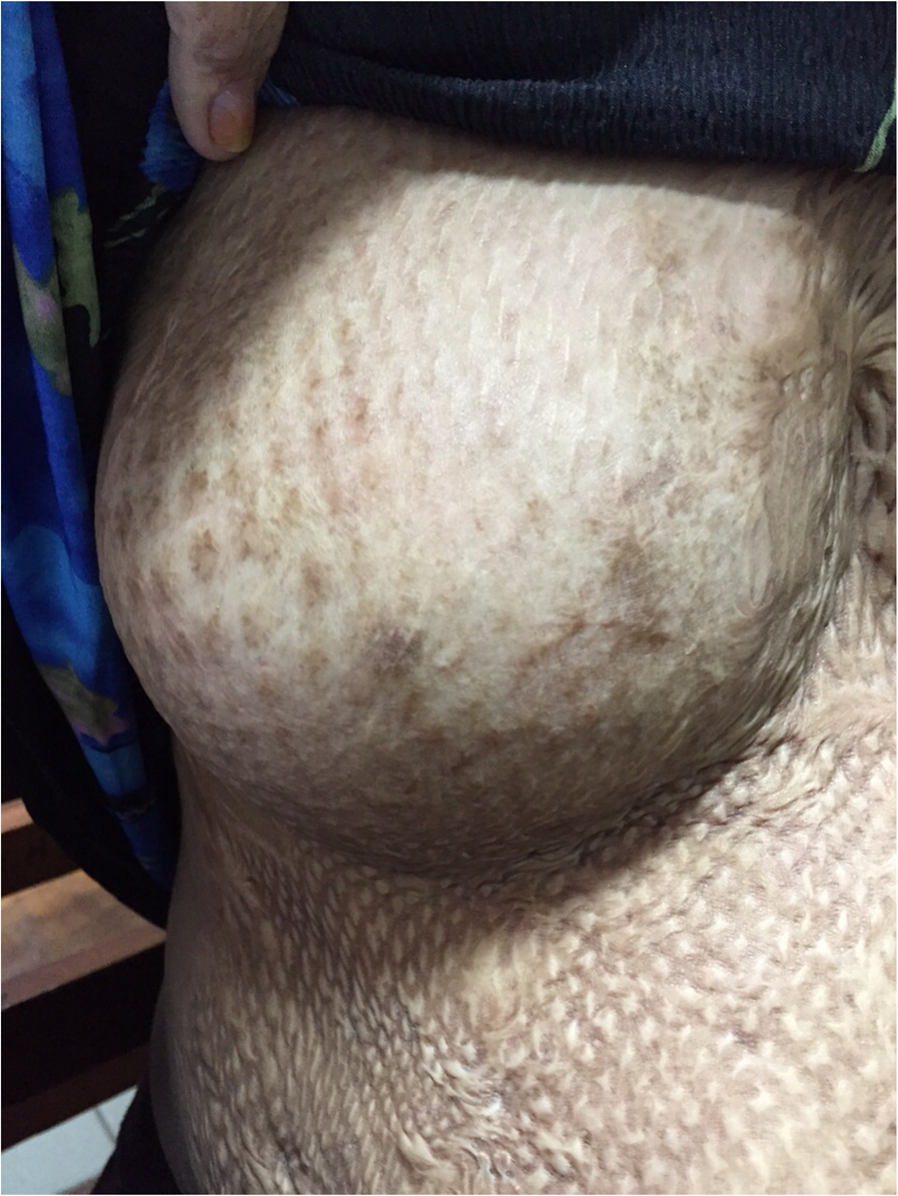
Antenatal: Right breast

**Fig. 2:**
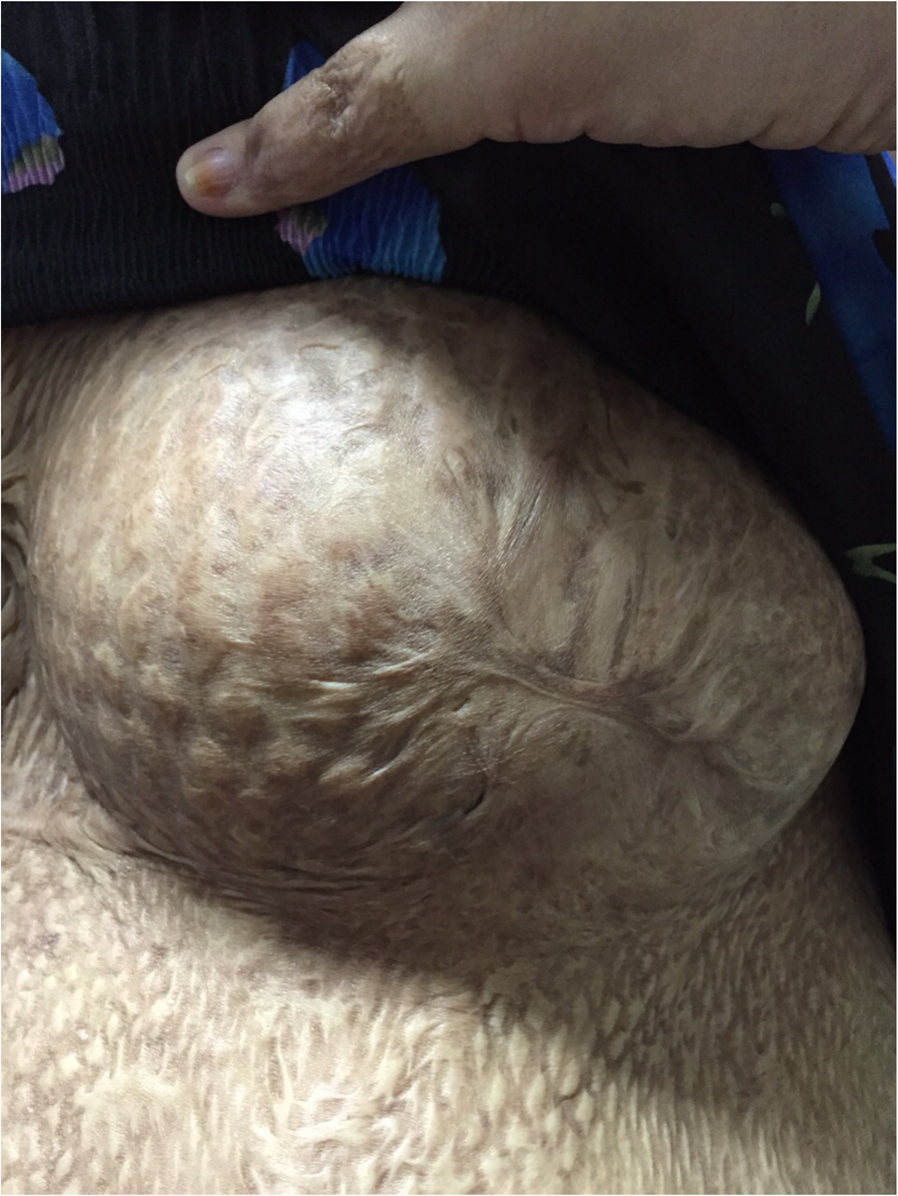
Antenatal: Left breast

**Fig. 3:**
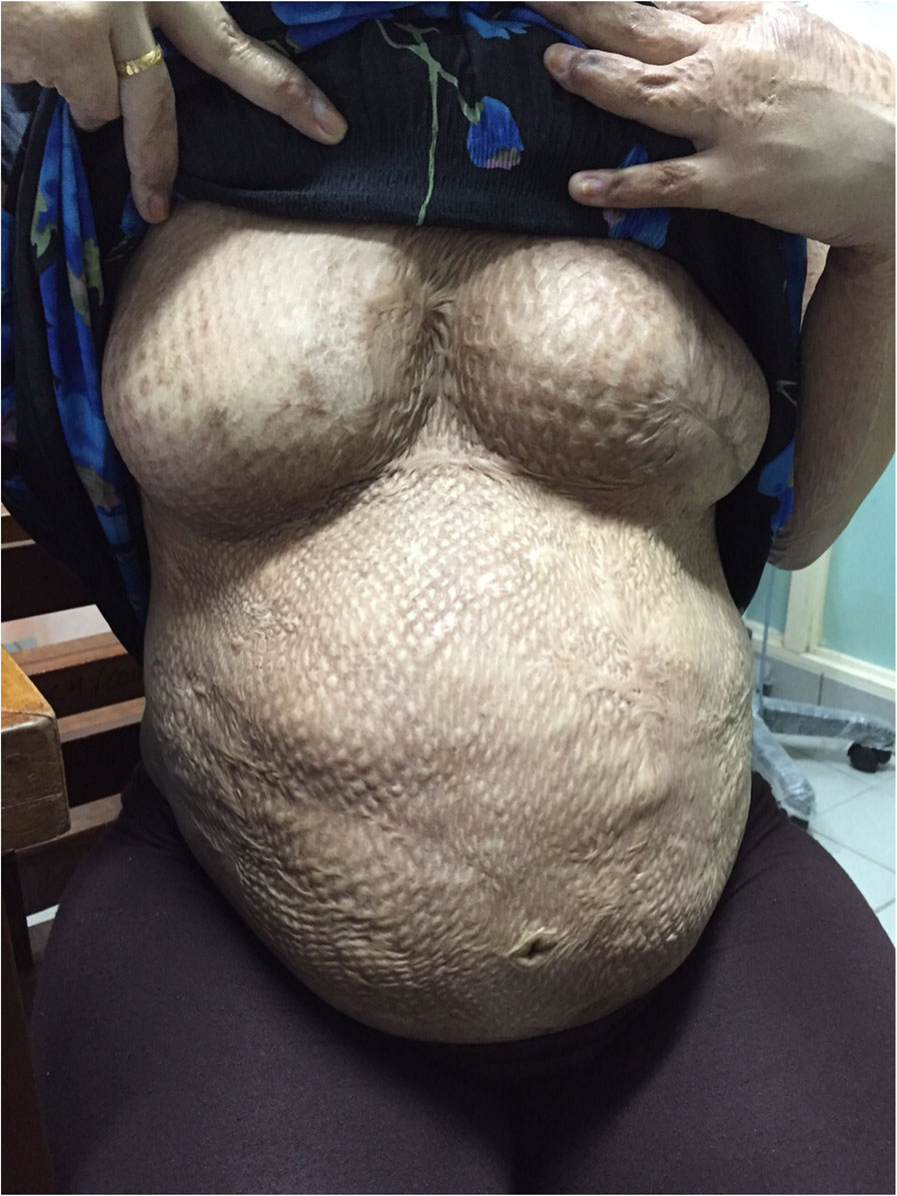
Antenatal: Breasts and abdomen

A young first time mother at 36 weeks period of gestation was seen in a health clinic in Kuching, Sarawak with regards to her ability to breastfeed. She was referred from a clinic in a small district located around 31 km from the nearest tertiary hospital. She had history of a flame burn at the age of 5 years old to her chest, abdomen, upper limb and part of her trunk. She was admitted for about 4 months in the Burn Unit in a tertiary hospital where skin grafts were done in 3 stages on her whole chest and abdomen. Despite the injuries, she noticed pubertal breasts development. During this pregnancy, she had further breast development which increased in size and her abdomen was able to stretch to accommodate her pregnancy. Physical examination showed a pregnancy which corresponded to her date. The skin over her chest and abdomen appeared tight with areas of hyperpigmentation and hypopigmentation due to scarring from the skin graft. There were normal breasts swelling and breast tissues palpable over her chest with the absence of areola and nipple. Sensations over the skin grafted area were intact. The breasts and abdomen (antenatally) are shown in Fig. 1 (Antenatal: Right breast), Fig. 2 (Antenatal: Left breast) and Fig. 3 (Antenatal: Breasts and abdomen).

### Management

A counselling session was conducted to explore her expectations and hopes regarding her ability to breastfeed her baby. Further counselling was done to prepare her that she might not be able to breastfeed her child due to her physical condition and may need lactation suppression post-birth. Alternative methods of feeding were discussed.

She birthed her infant at 36 weeks and 5 days via spontaneous vaginal delivery without any complications. Oral cabergoline 1 mg was given on the first postpartum day to suppress lactation. The baby was given infant formula via bottle feeding as the feeding method of choice. On subsequent days, she noticed her breasts reduced in size.

**Fig. 4:**
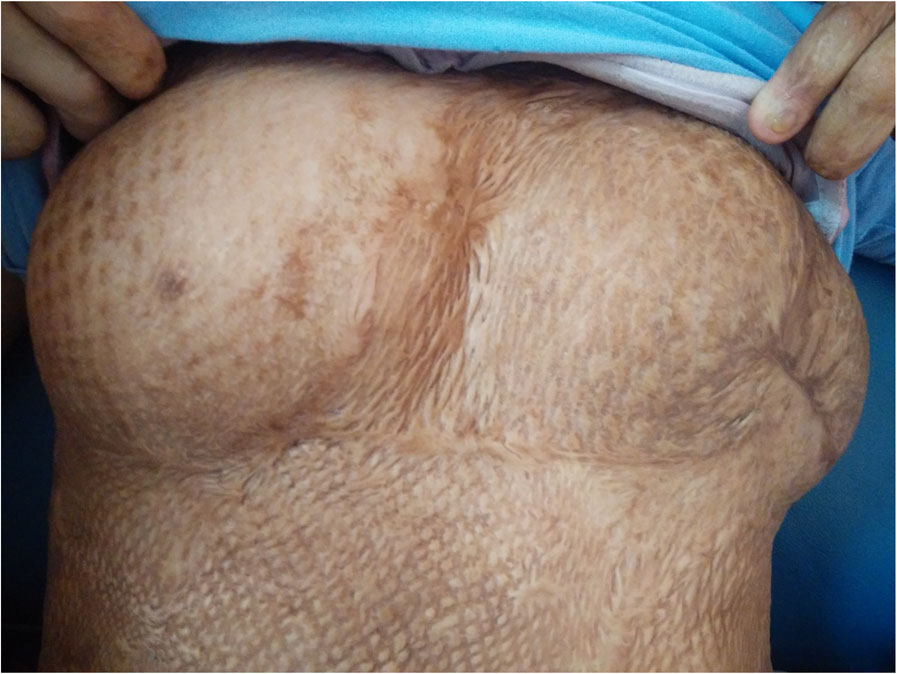
Postnatal Day 6: Breasts

At day 6 postpartum, her breasts reduced in size almost to the pre-pregnancy stage as shown in Fig. 4 (Postnatal Day 6: Breasts).

**Fig. 5:**
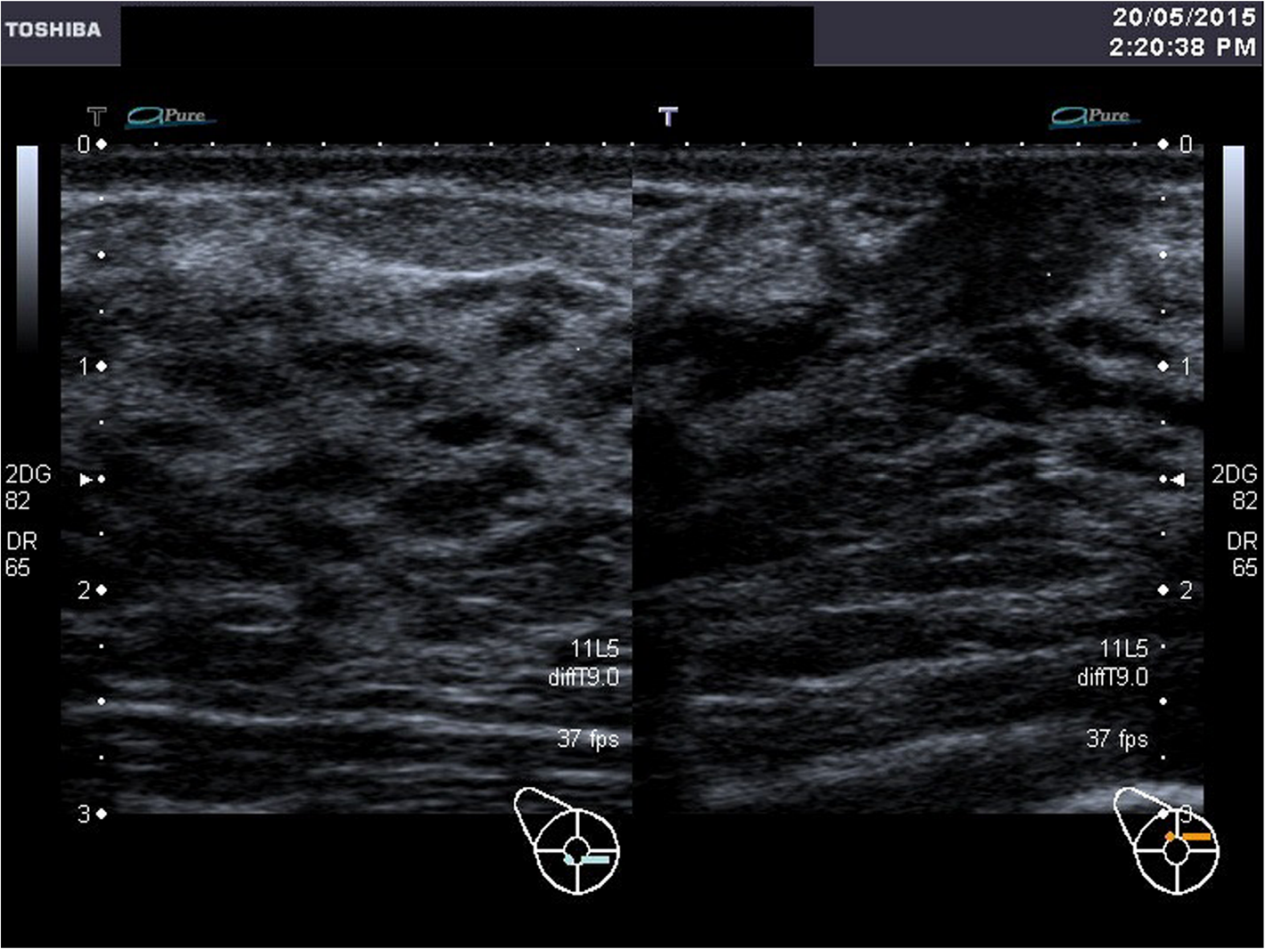
Ultrasound: Right breast

**Fig. 6:**
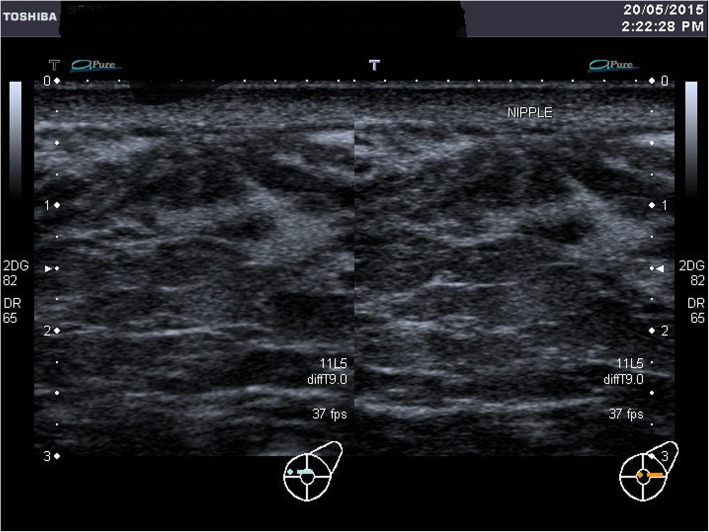
Ultrasound: Left breast

A breast ultrasound was done at day 6 postpartum. It showed dilated lactiferous ducts at retroareolar region bilaterally, consistent with lactating ducts of the breasts. The normally appearing vague shadow of the nipple was not seen. However, the tightly packed ducts seen parallel to the ultrasound beam are suggestive of major ducts to the possible location of the nipple as shown in Fig. 5 (Ultrasound: Right breast) and Fig. 6 (Ultrasound: Left breast).

A discussion with the plastic surgeon was made regarding the possibility of surgery to enable her to breastfeed in her future pregnancies. However, patient was not keen for any surgeries as she was made aware of the risks and complications. She also felt contented with her decision to bottle feed her child with infant formula.

Scheduled home postnatal visits were done to ensure that mother and baby were coping well. She had fully accepted that she was not able to breastfeed her child due to her physical limitations. Her husband and family were very understanding and supportive towards her. The baby was doing well with infant formula feeding. There were no signs and symptoms of postpartum depression.

## Discussion

Breastfeeding has been known for its benefit for the mother, child and society. Breastfeeding mothers who faced severe breastfeeding difficulties may feel lost in motherhood due to shattered expectations, a lost time for closeness and being no use to the infants [8]. Mothers who planned to breastfeed and continue to do so were less likely to have postpartum depression compared to mothers who planned to breastfeed but were not able to do so [9]. Thus, it is important to not only provide expert breastfeeding support to women who want to breastfeed; but also to provide compassionate support for mothers with intention to breastfeed, but who find themselves unable to [9].

The usage of supplemental nursing system for this mother-infant dyad could not be done, as the mechanism to attach the nursing tube to the breast needs the presence of the nipple. Thus, the infant was unable to latch due to the absence of the nipple. The mother was encouraged to bottle-feed the infant in a breastfeeding position as this action could increase the physical and emotional closeness between the mother and infant. Cup feeding was also taught to the mother as an alternative method to feed her infant. As part of the management to promote bonding and sense of motherhood, skin-to-skin contact immediately after delivery between mother and baby were encouraged. This practice was to continue in the subsequent care of the baby.

There are many reasons why a mother needs to suppress lactation. It can be due to personal choice to wean early and quickly, the baby may have died or they may need to stop breastfeeding due to medical reasons. This mother was keen to breastfeed her baby, but was not able to due to her physical limitations. Physiologically, mature breastmilk is made from about 36 to 96 h after delivery of the placenta (lactogenesis II) [10]. For most mothers, they will feel fullness of their breast and even tightness and soreness during this time. Breastmilk will be produced even if the baby does not suckle at the breast. Having breast engorgement with pain and discomfort in a mother who was not able to breastfeed and especially for this mother where there was no duct opening to the areolar for milk expression or leakage, would cause more discomfort which may affect her psychological wellbeing. Thus, lactation suppression was done to prevent this stage occurring.

Cabergoline is a dopaminergic receptor stimulant which inhibits prolactin secretion. For lactation inhibition to occur, a stat dose of 1 mg is given and 0.25 mg twice daily for two days for lactation suppression. A single 1 mg dose of cabergoline is at least as effective as bromocriptine 2.5 mg given twice daily for 14 days in suppressing lactation [11, 12]. With cabergoline, the incidence of rebound lactation in the third postpartum week is significantly lower [12]. Other drugs used conventionally for the inhibition and suppression of puerperal lactation are estrogens alone, estrogen-androgen combination and pyridoxine. However, the use of estrogens alone or with combination with androgen is no longer recommended due to its association with an increase incidence of postpartum thromboembolic disease and rebound lactation [13]. Cabergoline has been shown to be more effective for inhibition and suppression of lactation with the added advantage of easy dosage schedule and oral route of administration [14]. Thus, cabergoline was used for suppression of puerperal lactation in this mother. In this case, complete scarring of the nipple-areolar complex during her pre-pubescent stage following chest burns affected her opportunity to breastfeed her children. It was seen that her underlying immature breast tissue was not fully damaged as her breasts continued to develop without major distortion into adult breasts, even though the nipple-areolar complex is lost. If breastfeeding occurs, there may be problems arising due to decreased sensation during infant suckling, decreased tissue elasticity of the nipple and areola to form a teat and the scarring could affect maternal self-esteem, confidence and her ability to breastfeed which could hamper the milk-ejection reflex [15].

Counseling and providing support to mothers who intend to breastfeed but are unable to is beneficial in improving their mental health and reducing the risk of postpartum depression [9].

## Conclusions

In a mother who has breastfeeding difficulty particularly due to physical inability, counselling plays an important part in the management. Allaying the possible guilty feelings of not being able to breastfeed will fulfil the emotional gap which may arise in a mother with these challenges.

## References

[1] World Report on Child Injury Prevention (2008). Fact sheet. Children and Burns.

[2] Vijay K, Ramesh R, Sanjay MB (2012). Pediatric burn injuries. Int J Critical Illn Inj Sci.

[3] Foley P, Jeeves A, Davey RB, Sparnon AL (2008). Breast burns are not benign: long term outcomes of burns to the breast in pre pubertal girls. Burns.

[4] El-Otiefy MAE, Darwish AMA (2011). Post-burn breast deformity – various corrective techniques. Ann Burns Fire Disasters.

[5] Ogilvie MP, Panthaki ZJ (2008). Burns of the developing breast. J Craniofac Surg.

[6] Victora CG, Bahl R, Barros AJ, França GV, Horton S, Krasevec J, Murch S, Sankar MJ, Walker N, Rollins NC (2016). Breastfeeding in the 21st century: epidemiology, mechanisms, and lifelong effect. Lancet.

[7] Labbok M (2008). Exploration of guilt among mothers who do not breastfeed: the physician’s role. J Hum Lact.

[8] Palmer L, Carlsson G, Mollberg M, Nystrom M (2012). Severe breastfeeding difficulties: existential lostness as a mother - Women’s lived experiences of initiating breastfeeding under severe difficulties. Int J Qual Stud Health Well Being.

[9] Borra C, Iacovou M, Sevilla A (2015). New evidence on breastfeeding and postpartum depression: the importance of understanding women’s intentions. Matern Child Health J.

[10] Neville MC, Morton J (2001). Physiology and endocrine changes underlying human lactogenesis II. J Nutr.

[11] European Multicentre Study Group for Cabergoline in Lactation Inhibition (1991). Single dose cabergoline versus bromocriptine in inhibition of puerperal lactation: randomised, double blind, multicentre. Br J Med.

[12] Rains CP, Bryson HM, Fitton A (1995). Cabergoline. A review of its pharmacological properties and therapeutic potential in the treatment of hyperprolactinaemia and inhibition of lactation. Drugs..

[13] Kochenour NK (1980). Lactation suppression. Clin Obstet Gynecol.

[14] Singh N, Singh U, Sachan V (2009). Role of newer drug cabergoline in lactation suppression as compared to estrogen-androgen combination. J Obstet Gynecol India.

[15] Faridi MMA, Dewan P (2008). Successful breastfeeding with breast malformations. J Hum Lact.

